# Karyotypic diversity in four species of the genus *Gymnotus* Linnaeus, 1758 (Teleostei, Gymnotiformes, Gymnotidae): physical mapping of ribosomal genes and telomeric sequences

**DOI:** 10.3897/CompCytogen.v5i3.1375

**Published:** 2011-08-24

**Authors:** Priscilla Cardim Scacchetti, José Carlos Pansonato-Alves, Ricardo Utsunomia, Claudio Oliveira, Fausto Foresti

**Affiliations:** Laboratório de Biologia e Genética de Peixes, Instituto de Biociências de Botucatu, Universidade Estadual Paulista (UNESP), Departamento de Morfologia, Distrito de Rubião Junior, Botucatu, SP, Zip code: 18618-970 Brazil

**Keywords:** FISH, rDNA, cytogenetics, heterochromatin, chromosomal rearrangements

## Abstract

Conventional (Giemsa, C-Banding, Ag-NORs, CMA3) and molecular (5S rDNA, 18S rDNA, telomeric sequences) cytogenetic studies were carried out in specimens of ten distinct fish populations of the genus *Gymnotus* (*Gymnotus sylvius* Albert and Fernandes-Matioli, 1999, *Gymnotus inaequilabiatus* Valenciennes, 1839, *Gymnotus pantherinus* Steindachner, 1908, and *G*. cf. *carapo* Linnaeus, 1758) from different Brazilian hydrographic basins. *Gymnotus sylvius* presented a diploid number of 40 chromosomes (22m+12sm+6st), *Gymnotus pantherinus* presented 52 chromosomes (32m+18sm+2st), while *Gymnotus inaequilabiatus* (42m+10sm+2a)and *Gymnotus* cf. *carapo* (38m+12sm+4st) presented 54 chromosomes. The C-banding technique revealed centromeric marks in all chromosomes of all species. Besides that, conspicuous blocks of heterochromatin were found interstitially on the chromosomes of *Gymnotus inaequilabiatus*, *Gymnotus* cf. *carapo*,and *Gymnotus pantherinus*. All four species showed single nucleolus organizing regions confirmed by results obtained through Ag-NORs and FISH experiments using 18S rDNA probes, which showed the NORs localized on the first chromosome pair in *Gymnotus inaequilabiatus*, *Gymnotus* cf. *carapo*,and *Gymnotus pantherinus*, and on pair 2 in *Gymnotus sylvius*. CMA3 staining revealed additional unrelated NORs marks in *Gymnotus sylvius* and *Gymnotus pantherinus*. The 5S rDNA probes revealed signals on one pair in *Gymnotus sylvius* and two pairs in *Gymnotus pantherinus*; *Gymnotus inaequilabiatus* had about seventeen pairs marked, and *Gymnotus* cf. *carapo* had about fifteen pairs marked. It is considered that the high amount of heterochromatin identified in the chromosomes of *Gymnotus inaequilabiatus* and *Gymnotus* cf. *carapo* could have facilitated the dispersion of 5S rDNA in these species. Interstitial signals were detected on the first metacentric pair of *Gymnotus sylvius* by telomeric probes (TTAGGG)*n* indicating the possible occurrence of chromosomal fusions in this species. The present study reveals valuable cytotaxonomic markers for this group and allows a more precise evaluation of the processes involved in the karyotype differentiation and the interrelationships among different species of the genus *Gymnotus*.

## Introduction

Fish species belonging to the order Gymnotiformes, usually known as “tuviras”, “electric fish”, or “banded knife-fishes”, constitute a group endemic to the Neotropical region (Albert and Crampton 2003). This order holds more than 100 species and 27 genera that are grouped in five families: Gymnotidae, Rhamphichthyidae, Hypopomidae, Sternopygidae, and Apteronotidae (Mago-Leccia 1994). Among the Gymnotiformes, the karyotype diversity is better known in *Gymnotus* Linnaeus, 1758 and *Eigenmannia* Jordan and Evermann, 1896 genera (Albert and Crampton 2005).

Gymnotidae is currently composed of the genus *Gymnotus*, with 35 valid species, and *Electrophorus* Gill, 1864 with only one valid species (Froese and Pauly 2011). *Gymnotus* shows the widest geographic distribution in the group, occurring within inland waters of South and Central America, and is found from the Salado River, in the Argentinean “pampas” to San Nicolas River, Mexico, except Chile and Belize (Albert et al. 2005). The genusis more diversified in the Amazon River basin, where 19 species are known, including species not formally described (Crampton et al. 2005, Froese and Pauly 2011).

The available cytogenetics data for *Gymnotus* species evidence a high karyotypic diversity characterized by different diploid numbers observed in some species, as in *Gymnotus carapo* Linnaeus, 1758 and *Gymnotus inaequilabiatus* Valenciennes, 1839 with 54 chromosomes; *Gymnotus sylvius* Albert and Fernandes-Matioli, 1999 which shows 40 chromosomes; *Gymnotus pantherinus* Steindachner, 1908 with 52 chromosomes, and *Gymnotus capanema* Milhomem et al. in press with 34 chromosomes, the smallest diploid number observed for this genera so far (reviews: Margarido et al. 2007, Milhomem et al. in press). *Gymnotus pantanal* Fernandes-Matioli et al., 2005 presents 40 chromosomes in females and 39 in males, suggesting the occurrence of a multiple sex chromosome system in this species (Silva and Margarido 2005).

The current study was carried out aiming to broaden the cytogenetic data available for *Gymnotus*, mapping the distribution of ribosomal sites and telomeric DNA sequences on the chromosomes of different species of this genus. The data obtained will allow a better understanding of the mechanisms involved in the process of karyotypic differentiation and diversification of this fish group.

## Material and methods

**Figure 1.**
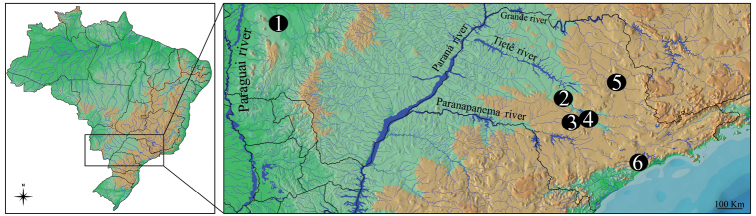
Map of Brazil showing the collection sites of species and populations of *Gymnotus* analyzed. **1** Miranda River, Passo do Lontra – MT, *Gymnotus* cf. *carapo* **2** Campo Novo River, Bauru – SP, *Gymnotus sylvius* and *Gymnotus inaequilabiatus* **3** Água da Madalena River, Botucatu – SP, *Gymnotus sylvius* and *Gymnotus inaequilabiatus* **4** Araquá River, Botucatu – SP, *Gymnotus sylvius* and *Gymnotus inaequilabiatus* **5** Mogi-Guaçu River, Pirassununga – SP, *Gymnotus sylvius* and *Gymnotus inaequilabiatus*; 6. Aguapeú River, Mongaguá – SP, *Gymnotus pantherinus*.

Four fish species of *Gymnotus* sampled throughout the different components of the Brazilian hydrographic river basins were cytogenetically analyzed (Figure 1 and Table 1). After analysis, the specimens were deposited in the fish collection of the Laboratório de Biologia e Genética de Peixes (LBP), Universidade Estadual Paulista, at Botucatu, São Paulo, Brazil.

**Table 1.** Specimens of *Gymnotus* analyzed. LBP – deposit voucher number at the fish collection of the Laboratório de Biologia e Genética de Peixes, Instituto de Biociências de Botucatu, UNESP. F – females, M – males.

Species	LBP	Sample Localities	F	M	Coordinates
*Gymnotus sylvius*	11160	Água da Madalena - Botucatu-SP River	20	14	S22°59.25', W48°25.40'
*Gymnotus sylvius*	11155	Araquá – Botucatu-SP River	02	-	S22°47.13', W48°28.89'
*Gymnotus sylvius*	11163	Campo Novo- Bauru-SP River	01	01	S22°23.07', W49°00.55'
*Gymnotus sylvius*	11161	Mogi-Guaçu - Pirassununga-SP River	-	01	S21°55.50', W47°22.29'
*Gymnotus inaequilabiatus*	11154	Água da Madalena - Botucatu-SP River	02	07	S22°59.25', W48°25.40'
*Gymnotus inaequilabiatus*	11158	Araquá – Botucatu-SP River	04	02	S22°47.13', W48°28.89'
*Gymnotus inaequilabiatus*	11152	Campo Novo - Bauru-SP River	06	13	S22°23.07', W49°00.55'
*Gymnotus inaequilabiatus*	11156	Mogi-Guaçu - Pirassununga-SP River	06	17	S21°55.50', W47°22.29'
*Gymnotus pantherinus*	11153	Aguapeú - Mongaguá-SP River	03	02	S24°06.40', W46°43.00'
*Gymnotus* cf. *carapo*	9836	Miranda - Pantanal-MSRiver	03	02	S19°34.34', W57°02.17'

The fishes were euthanized with a lethal dose of benzocaine before the procedures of chromosome preparation. Mitotic chromosome preparations were carried out according to Foresti et al. (1993). The nucleolus organizer regions (NORs) were localized on chromosomes by silver nitrate staining, according to Howell and Black (1980), and C-banding patterns were obtained following the protocol described by Sumner (1972).

Molecular cytogenetic analysis involved the use of GC-specific fluorochrome Chromomycin A3 (CMA3) (Schweizer 1976) and probes of specific gene sequences. Fluorescent *in situ* hybridization was carried out to locate the rDNA genes on chromosomes, according to the procedure established by Pinkel et al.(1986) using stringency of 77%. The 18S rDNA probes were obtained by PCR (Polymerase Chain Reaction) from total DNA of *Gymnotus* cf. *carapo* using primers NS1 5’-GTAGTCATATGCTTGTCTC-3’ and NS8 5’-TCCGCAGGTTCACCTACGGA-3’ (White et al. 1990) and the 5S rDNA probes from total DNA of *Synbranchus marmoratus* Bloch, 1795 using the primers 5SA (5’- TACGCCCGATCTCGTCCGATC-3’) and 5SB (5’-GCTGGTATGGCCGTAGC-3’) (Martins and Galetti Jr 1999). The 18S rDNA probe in *Gymnotus pantherinus* and 5S rDNA probes in *Gymnotus sylvius*, *Gymnotus* cf. *carapo* and *Gymnotus inaequilabiatus* were labeled with digoxigenin-11-dUTP (Roche Applied Science)by PCR and the detection of hybridization signs was obtained with anti-digoxigenin-rhodamine (Roche Applied Science). The 5S probe in *Gymnotus pantherinus* and 18S rDNA probes in *Gymnotus sylvius*, *Gymnotus* cf. *carapo* and *Gymnotus inaequilabiatus* were labeled with biotin-16-dUTP (Roche Applied Science) by PCR and the detection of hybridization signs with avidin-FITC. Telomeric sites were identified with probes for sequences (TTAGGG)5 and (CCCTAA)5 labeled with digoxigenin 11-dUTP (Roche Applied Science) and the hybridization signs were detected with anti-digoxigenin-rhodamine. Chromosome morphology was determined according to arm relationships proposed by Levan et al. (1964), and the chromosomes were arranged in decreasing size order in the karyotypes.

## Results

**Figure 2a–d.**
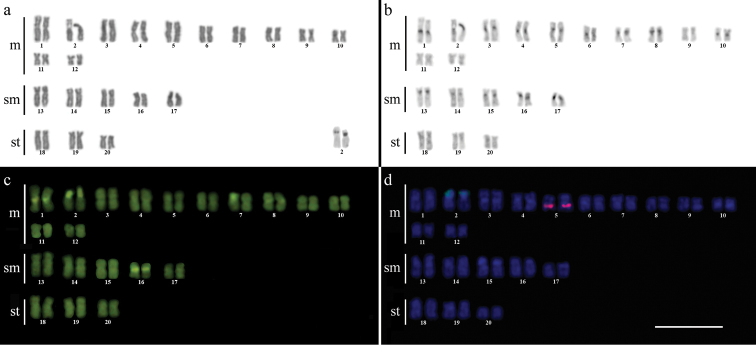
Karyotype of *Gymnotus sylvius* after (**a**) conventional Giemsa staining, (**b**) C-banding, (**c**) CMA3 fluorochrome staining, (**d**) double FISH with 5S rDNA (red) and 18S rDNA (green) probes. Bar = 10µm.

**Figure 3a–d.**
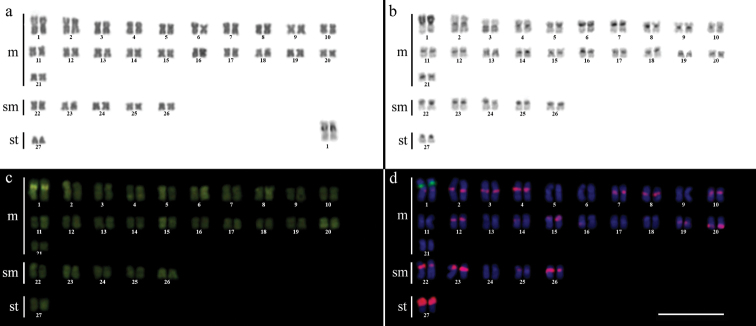
Karyotype of *Gymnotus inaequilabiatus* after (**a**) conventional Giemsa staining, (**b**) C-banding, (**c**) CMA3 fluorochrome staining, (**d**) double FISH with 5S rDNA (red) and 18S rDNA (green) probes. Bar = 10µm.

**Figure 4a–d.**
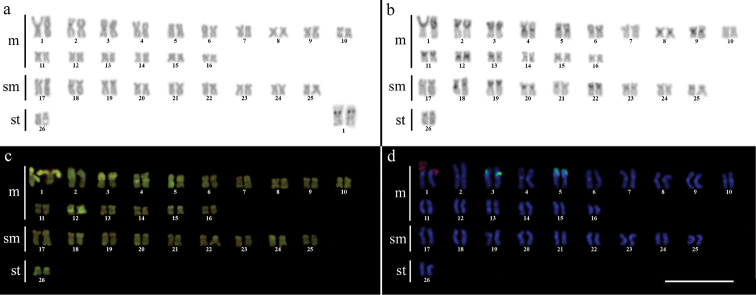
Karyotype of *Gymnotus pantherinus* after (**a**) conventional Giemsa staining, (**b**) C-banding, (**c**) CMA3 fluorochrome staining, (**d**) double FISH with 5S rDNA (green) and 18S rDNA (red) probes. Bar = 10µm.

**Figure 5a–d.**
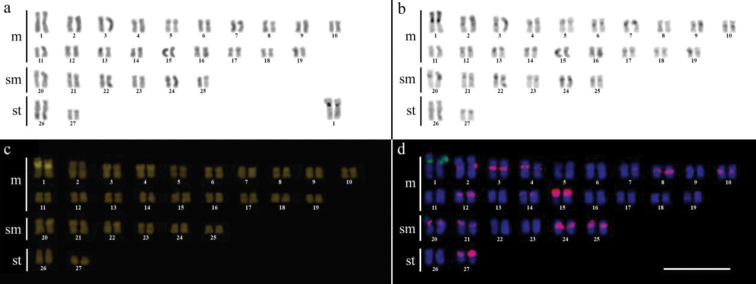
Karyotype of *Gymnotus* cf. *carapo* after (**a**) conventional Giemsa staining, (**b**) C-banding, (**c**) CMA3 fluorochrome staining, (**d**) double FISH with 5S rDNA (red) and 18S rDNA (green) probes. Bar = 10µm.

Cytogenetic analysis performed in representatives of four *Gymnotus* fish species evidenced an expressive variation in the diploid number among the species, despite the conservative karyotypic feature among the representatives of the populations. *Gymnotus sylvius* presented 40 chromosomes (Fig. 2a); *Gymnotus inaequilabiatus* and *Gymnotus* cf. *carapo* presented 54 chromosomes (Figs 3a, 5a), and *Gymnotus pantherinus* presented 52 chromosomes (Fig. 4a). Data are summarized in Table 2.

**Table 2.** Cytogenetic data on four species of *Gymnotus*. ITS – Interstitial Telomeric Sites; (I) – Interstitial mark.

Species	5S rDNA	18S rDNA	ITS	CMA3	Karyotypic formulae
*Gymnotus sylvius*	Pair 4	2 (I)	Pair 1	Pairs 1,2 and 16	22m+12sm+6st
*Gymnotus inaequilabiatus*	Up to17 pairs	1 (I)	--	Pair 1	42m+10sm+2a
*Gymnotus pantherinus*	Pairs 3 and 5	1 (I)	--	Pairs 1,3, 4 and 12	32m+18sm+2st
*Gymnotus* cf. *carapo*	Up to 15 pairs	1 (I)	--	Pair 1	38m+12sm+4st

The C-banding technique revealed significant differences in the distribution patterns of heterochromatin among the analyzed species. All populations of *Gymnotus sylvius* showed small amounts of constitutive heterochromatin restricted to the centromeric areas of all chromosomes, and also blocks coincident with NORs (Fig. 2b). In *Gymnotus inaequilabiatus* (Fig. 3b), *Gymnotus pantherinus* (Fig. 4b) and *Gymnotus* cf. *carapo* (Fig. 5b), besides centromeric and pericentromeric marks, it was possible to observe conspicuous interstitial blocks of heterochromatin in some chromosomes. No numerical or structural polymorphisms related to the presence of supernumerary or sex chromosomes were detected in the samples analyzed.

The impregnation by silver nitrate evidenced that all species and populations of *Gymnotus* analyzed hold a simple pair of chromosomes bearing NORs. The populations of *Gymnotus sylvius* showed signalsat the interstitial region on the short arms of chromosome pair 2 (highlighted in Fig. 2a). The representatives of the other species showed their ribosomal sites located in the interstitial position on the short arms of chromosome pair number 1 (highlighted in Figs 3a–5a). The use of 18S rDNA probe confirmed the results achieved with silver nitrate staining (Figs 2d–5d), while the hybridization with 5S rDNA probes localized this gene in the pericentromeric position of pair number 4 in the representatives of *Gymnotus sylvius* populations (Fig. 2d); in two chromosome pairs (numbers 3 and 5) in *Gymnotus pantherinus* (Fig. 4d); in up to 17 chromosomal pairs in the representatives of *Gymnotus inaequilabiatus*, and in up to 15 pairs in *Gymnotus* cf. *carapo* (Figs 3d, 5d). The coloration with fluorochrome CMA3 in *Gymnotus inaequilabiatus* and *Gymnotus* cf. *carapo* marked only the pair bearing the NORs (Figs 3c, 5c), while *Gymnotus sylvius* and *Gymnotus pantherinus* showed additional marked pairs besides the chromosomes bearing NORs (Figs 2c, 4c).

**Figure 6a–d.**
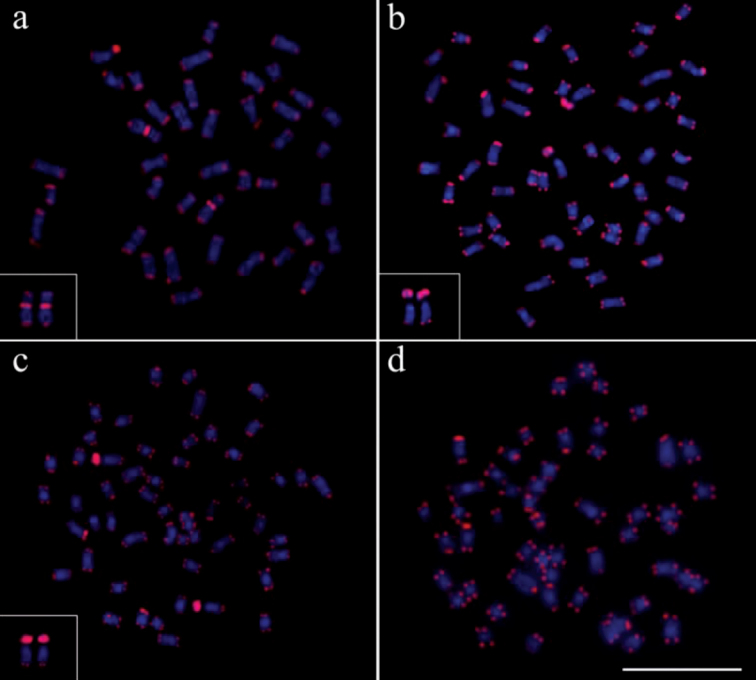
Distribution pattern of telomeric sites in metaphases of the representatives of the four species of *Gymnotus* analyzed. (**a**) *Gymnotus sylvius* (featured interstitial telomeric sites – ITS), (**b**) *Gymnotus inaequilabiatus*, (**c**) *Gymnotus* cf. *carapo* and (**d**) *Gymnotus pantherinus*. Bar = 10µm.

**Figure 7a–d.**
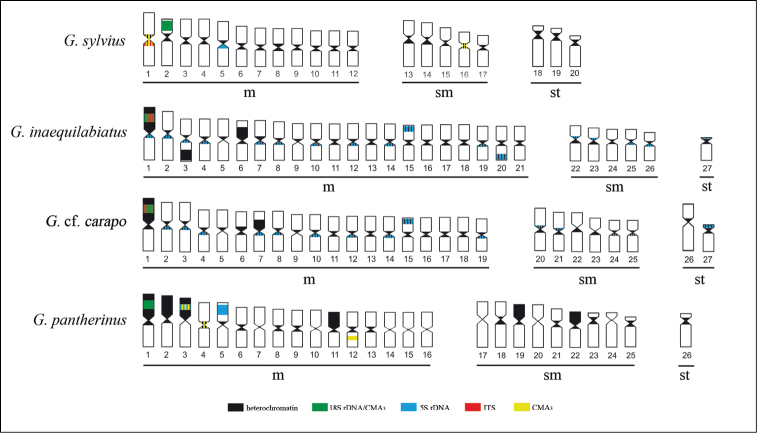
Ideogram showing the hybridization patterns described in this paper. The overlapping signals are represented simultaneously by the respective colors (**a**) *Gymnotus sylvius*, (**b**) *Gymnotus inaequilabiatus*, (**c**) *Gymnotus* cf. *carapo* and (**d**) *Gymnotus pantherinus*.

The use of telomeric probes (TTAGGG)n evidenced signals in the terminal position of all chromosomes in all populations analyzed (Fig. 6). Additionally, conspicuous marks were found along the nucleolar regions in the specimens of *Gymnotus inaequilabiatus* (Fig. 6b) and *Gymnotus* cf. *carapo* (Fig. 6c). Besides that, interstitial telomeric sites (ITS) were observed in the chromosomes of pair number 1 in *Gymnotus sylvius* (Fig. 6a). All data are summarized in Table 2 and represented in an ideogram in Figure 7.

## Discussion

Available cytogenetic data on the genus *Gymnotus* evidence the occurrence of high karyotypic diversity among the species, notably related to diploid number and karyotypic formulae, ranging from 34 chromosomes in *Gymnotus capanema* up to 54 chromosomes in *Gymnotus inaequilabiatus* (Fernandes-Matioli et al. 1998, Milhomem et al. in press). In the present work, cytogenetic analysis performed in individuals of different populations of four species of *Gymnotus* confirmed the chromosomal variability, evidencing the occurrence of notable differences among the karyotypes of different species. However, a striking conservation of karyotypic features was observed among the different populations analyzed, mainly among populations of *Gymnotus sylvius* and *Gymnotus inaequilabiatus*.

The karyotype diversity found in the species may be related to the fact that the representatives of *Gymnotus* are generally endemic organisms living in headwaters, which do not migrate long distances. Such characteristic may act to reduce gene flow among different populations, even in the same hydrographic basin, resulting in differences between populations of the same species, as found in samples of *Characidium* Reinhardt, 1867 (Pansonato-Alves et al. 2010, 2011a). However, no significant differences were detected among the karyotypes in the different populations of the species analyzed. Despite the short-distance migratory behavior, the species of the genus *Gymnotus* are widely distributed throughout the Neotropical region and inhabit a wide diversity of environments, ranging from systems of flow rivers to flood plains (Albert et al. 2005, Albert and Crampton 2005). Thus, during the rainy seasons, the representatives of *Gymnotus* inhabiting flood plains could change location by passive dispersal, migrating from one part of the river to another favoring the maintenance of gene flow among populations of different river systems, as may have occurred among populations of *Hoplias malabaricus* Bloch, 1794 (Blanco et al. 2010).

The karyotypic identity observed among populations inside the species of *Gymnotus* reinforces the postulate that cytogenetic characteristics could be considered an important tool for taxonomic diagnostic of species in this fish group (Margarido et al. 2007).

The differences related to the number and morphology of the chromosomes found among the species suggest the occurrence of structural and numerical rearrangements during the process of differentiation. Milhomem et al. (2008) detected possible alterations in karyotype structure in representatives of *Gymnotus carapo* with 40 and 42 chromosomes from the Amazon river basin, while Claro and Almeida-Toledo (2010) found this same situation in *Gymnotus sylvius* (2n=40) and *Gymnotus* cf. *carapo* (2n=54) from the Paraná river basin. These authors suggested that the differences found in the diploid number of these species might have arisen from chromosomal rearrangements, mainly centric fusions. Notwithstanding these authors’ proposition, using whole chromosomes probes in the same sample analyzed by Milhomem et al. (2008), Nagamachi et al. (2010) established that the structural modifications found in the karyotypes could be far more complex than a result of simple fusion or centric fission events. The analysis also revealed that the representatives of the genus *Gymnotus* showed high genomic plasticity, and that the analyzed samples from the Amazon basin, usually denominated cryptic, were, in fact, distinct species.

In the current study, the probes used for telomeric sequence (TTAGGG)n revealed signals of hybridization on the extremities of all chromosomes in all populations analyzed (Figure 6). However, interstitial telomeric sites (ITSs) were observed in the chromosomes of *Gymnotus sylvius*. The presence of these ITSs in some chromosomes could be an indicative of recent centric fusion events, as previously discussed by Claro and Almeida-Toledo (2010) in *Gymnotus sylvius* and *Gymnotus* cf. *carapo* and Milhomem et al. (2008) in *Gymnotus carapo*. These authors proposed that chromosomal fusion events would act as the most important mechanisms of karyotype evolution in this fish group. Further studies by Claro and Almeida-Toledo (2010), using 5-BrdU incorporation in the study of *Gymnotus sylvius* and *Gymnotus* cf. *carapo* chromosomes, detected homologies among multiple chromosomes in these species, with a complete correspondence of bands, indicating a probable common ancestral origin.

The occurrence of ITS in some chromosomes of *Gymnotus sylvius*, as well as its absence in *Gymnotus pantherinus*, could be attributed to different factors, such as the occurrence of differences in the type of chromosomal rearrangements, the plasticity of the telomeric sequences or the divergence time of the species, which originated modifications in the sequences and possibly made them undetectable by the FISH technique. In a phylogenetic reorganization of the Gymnotiformes based on molecular and cytogenetic data, Fernandes-Matioli and Almeida-Toledo (2001) suggested that *Gymnotus sylvius* constitutes the most derived species amongst the representatives of *Gymnotus*, while *Gymnotus pantherinus* apparently differentiated much longer ago. The presence of the ITS in *Gymnotus sylvius* and its absence in *Gymnotus panth*e*rinus* could be justified by the divergence time between these species. Furthermore, considering that a karyotype presenting 52 chromosomes would characterize the basal genomic group for *Gymnotus* (Fernandes-Matioli and Almeida-Toledo 2001), it could be expected that more chromosomes would present ITS in the karyotype of *Gymnotus sylvius*. Thence, the occurrence of only one pair of chromosomes with ITS in this species could be related to later modifications occurred in these sites, making it impossible to be detected by conventional FISH. Such situation is also proposed to occur in *Gymnotus capanema* (2n=34), a species with the smallest numer modifications occurred in these sites, making it impossible to be detected by conventional FISH. Such situation is also proposed to occur in *Gymnotus capanema* (2n=34), a species with the smallest number of chromosomes within the genus *Gymnotus* with no ITS detected (Milhomem et al. in press). Another possible explanation to the absence of extra ITS in *Gymnotus sylvius* would be caused by a loss of the telomere repetition, which could have facilitated events of chromosome fusion (Blasco et al. 1997). This hypothesis also helps to explain why not all fused chromosomes have interstitial telomeric sites.

The identification of nucleolus organizer regions in the four species analyzed through silver nitrate staining and 18S rDNA probes revealed only one chromosome pair containing nucleolar sites and characterizing a simple NORs system, as previously cited (Fernandes-Matioli et al. 1997). The polymorphism in the size of the NOR sites among homologous chromosomes, which is commonly found in fish, was also detected in the species of the genus *Gymnotus* (Foresti et al. 1981, Fernandes-Matioli et al. 1997). These results indicate the conservatism of NORs in this group, not only for its location, generally on the first pair of chromosomes in multiple species studied, but also for its occurrence in only one pair of chromosomes, which characterizes a simple NORs system, a situation also found in other fish groups, such as the cichlids (Feldberg et al. 2003). Withal, analyzing three sympatric species of *Gymnotus*, the motile and dynamic character of these sites was confirmed and permitted identification species-specific Ag-NORs marks, which led the authors to consider this feature as an interesting cytotaxonomic tool (Lacerda and Maistro 2007).

The use of CMA3 in metaphase chromosomes revealed additional marks to those identified in the ribosomal sites in *Gymnotus sylvius* and *Gymnotus pantherinus*,indicating the presence of additional GC-rich sequences. Gold and Amemiya (1986) affirmed that the treatment with CMA3 would mark active and inactive ribosomal sites. Nevertheless,the presence of additional GC-rich sequences in chromosomes without ribosomal cístrons in *Gymnotus sylvius* and *Gymnotus pantherinus* indicates heterochromatin heterogeneity between these species. The heterochromatin is mainly composed of repetitive DNA sequences, which is thought to evolve in parallel (Dover 1986), resulting in homogenization of sequences within species (Ugarkovic and Plohl 2002). In this way, the distinct heterochromatin differentiation processes in different species of *Gymnotus* could have originated such patterns of CMA3 staining.

The distribution patterns of 5S rDNA sequences showed a peculiar dispersion of these repetitive sites in the karyotypes of the species in the genus *Gymnotus*. Considering the great blocks of heterochromatin and the variation in the distribution pattern of 5S rDNA sites in *Gymnotus inaequilabiatus*, *Gymnotus* cf. *carapo*, and even in *Gymnotus pantherinus*, it can be considered that this situation might have favored the occurrence of structural rearrangements in the karyotype of these species, since heterochromatic areas are more propitious to breaks, and thus may facilitate the dispersion of this gene sequence. The absence of large heterochromatic blocks and the presence of 5S rDNA in a unique pair of chromosomes in *Gymnotus sylvius* could reinforce this hypothesis. Martins and Galletti (2001) related the existence of two classes of 5S rDNA located in different chromosomes of fish belonging to *Leporinus* Spix, 1829 genus. Considering the high degree of dispersion of 5S rDNA in *Gymnotus inaequilabiatus* and *Gymnotus* cf. *carapo* species, it is possible that different classes of this ribosomal gene might be differentially distributed in the chromosomes of this species. On the other hand, in *Gymnotus pantherinus* and *Gymnotus sylvius*, the distinct classes of 5S rDNA could be co-located in the same chromosomes.

Recent studies carried out by Cioffi et al. (2010) suggest that transposable elements of *Rex3* retrotransposon type might be associated to the distribution and dispersion of 5S rDNA in the karyotypes of species in the genus *Erythrinus* Bloch and Schneider, 1801. However, the existence of pseudogenes in the genome of *Gymnotus inaequilabiatus* and *Gymnotus* cf. *carapo* cannot be discarded, since the heterochromatin could have sequences similar to those of 5S rDNA, as suggested to occur in fish belonging to the genus *Characidium* (Pansonato-Alves et al. 2011b) and *Centropyge* Kaup, 1860 (Affonso and Galetti 2005).

Despite the marked karyotypic conservation found inside the populations of *Gymnotus* species, the results achieved in the current work revealed great differences in the chromosome structure in the species of this genus, indicating that all possible evolution ways passed through the differentiation process of chromosomes.

## Appendix 1

Nexus file of aligned COI and COII nucleotide sequences. File format: Nexus file. (nxs).
